# Caffeic Acid Phenethyl Ester (CAPE) Induced Apoptosis in Serous Ovarian Cancer OV7 Cells by Deregulation of BCL2/BAX Genes

**DOI:** 10.3390/molecules25153514

**Published:** 2020-07-31

**Authors:** Anna Kleczka, Robert Kubina, Radosław Dzik, Krzysztof Jasik, Jerzy Stojko, Krzysztof Cholewa, Agata Kabała-Dzik

**Affiliations:** 1 Department of Pathology, Faculty of Pharmaceutical Sciences in Sosnowiec, Medical University of Silesia in Katowice, Ostrogórska 30, 41-200 Sosnowiec, Poland; akleczka@sum.edu.pl (A.K.); rkubina@sum.edu.pl (R.K.); kjasik@sum.edu.pl (K.J.); 2 Department of Biosensors and Processing of Biomedical Signals, Faculty of Biomedical Engineering, Silesian University of Technology, Roosevelta 40, 41-800 Zabrze, Poland; radoslaw.dzik@gmail.com; 3 Department of Toxicology and Bioanalysis, Faculty of Pharmaceutical Sciences in Sosnowiec, Medical University of Silesia in Katowice, Jagiellońska 4, 41-200 Sosnowiec, Poland; jstojko@sum.edu.pl; 4 Department of Biochemistry, Faculty of Pharmaceutical Sciences in Sosnowiec, Medical University of Silesia in Katowice, Jedności 8B, 41-200 Sosnowiec, Poland; kcholewa@sum.edu.pl

**Keywords:** caffeic acid phenethyl ester, CAPE, ovarian cancer, OV7, cytotoxicity, apoptosis

## Abstract

Ovarian cancer has the worst prognosis among all gynecological cancers. Therefore, it seems reasonable to seek new drugs that may improve the effectiveness of treatment or mitigate the adverse effects of chemotherapy. Caffeic acid phenethyl ester (CAPE) has many beneficial biological properties. The aim of the study was to assess the anticancer properties of CAPE against serum ovarian carcinoma cells. The morphology of the cells was evaluated in H-E staining and in transmission electron microscopy. The cytotoxic and proapoptotic activity of CAPE was investigated by using the XTT-NR-SRB assay, qRT-PCR analysis of BAX/BCL2 expression, and by cytometric evaluation. CAPE causes constriction in OV7 cells, numerous granulomas were observed in the cytoplasm, the cell nuclei were pyknotic. Autophagosomal vacuoles could suggest the occurrence of aponecrosis. CAPE significantly decreased the lysosomal activity and the total synthesis of cellular proteins. CAPE exhibited, dose and time dependent, cytotoxic activity against OV7 serum ovarian cancer cells. In OV7 cells CAPE induced apoptosis via dysregulation of BAX/BCL2 balance, while activated proapoptotic BAX gene expression level was 10 times higher than BCL2.

## 1. Introduction

Caffeic acid phenethyl ester (CAPE) is a bioactive compound produced as a secondary metabolite by most plants. Literature data indicate that CAPE presents a number of antimicrobial, antioxidant, anti-inflammatory, immunomodulatory and cytotoxic properties [1,2,3]. Researchers have also proven that CAPE is a therapeutically versatile polyphenol and an effective chemotherapy adjuvant. The administration of CAPE in order to increase therapeutic efficacy and reduce chemotherapy-induced toxicity may find wide application in the treatment of many cancers [4].

The mechanism of CAPE antitumor activity is based on the inhibition of NF-κB (nuclear factor kappa-light-chain-enhancer of activated B cells). NF-κB is a protein transcription factor that affects the activity of crucial proto-oncogenes and promotes the growth and survival of altered cells during the cell cycle [5,6].

The broad spectrum of biological activities of CAPE can support oncological treatment. The effects of CAPE on prostate cancer, breast cancer, head and neck cancers, liver, pancreatic, colorectal and ovarian cancer have been studied [7,8].

Ovarian cancer is the seventh most common cancer among women in the world and has the highest risk of death among all gynecological cancers. Malignant ovarian tumors are usually detected late in advanced clinical stages [9]. Statistics show that malignant ovarian tumors are only in 17% of cases limited to the organ, at the time of detection. Over 62% of patients with ovarian cancer are diagnosed after distant metastases occurrence [10].

The standard treatment of ovarian cancer involves the use of three complementary methods: surgery, chemotherapy and radiation. The effectiveness of these therapeutic procedures depends, among others on the patient’s general health, histological type and stage of cancer. Ovarian cancer is very sensitive to chemotherapy. Unfortunately, despite the initially good response to the treatment, many patients relapse, which requires multidrug therapy and is very burdensome for the body [11,12]. Extensive research is underway to confirm the possibility of using phytotherapy in the treatment of ovarian cancer [13].

The direct goal of this study is to assess the antiproliferative, cytotoxic and proapoptotic properties of CAPE in the treatment of ovarian cancer OV7 cells. To date, studies on the effect of CAPE on poorly differentiated, serous ovarian cancer OV7 cells have not been conducted. The research conducted so far was focused on assessing the effects of flavonoids and polyphenols, and their derivatives on ovarian adenocarcinoma. The serous type of ovarian cancer occurs most often and is diagnosed at a late stage of clinical advancement; therefore, it seems important to conduct the research aimed at discovering new therapeutic possibilities for ovarian serous cancer. The proapoptotic effect of CAPE on cancer cells seems particularly important.

## 2. Results

### 2.1. Microscopic Evaluation of OV7 Cells Morphology in Hematoxylin and Eosin Staining Protocol

**Figure 1:**
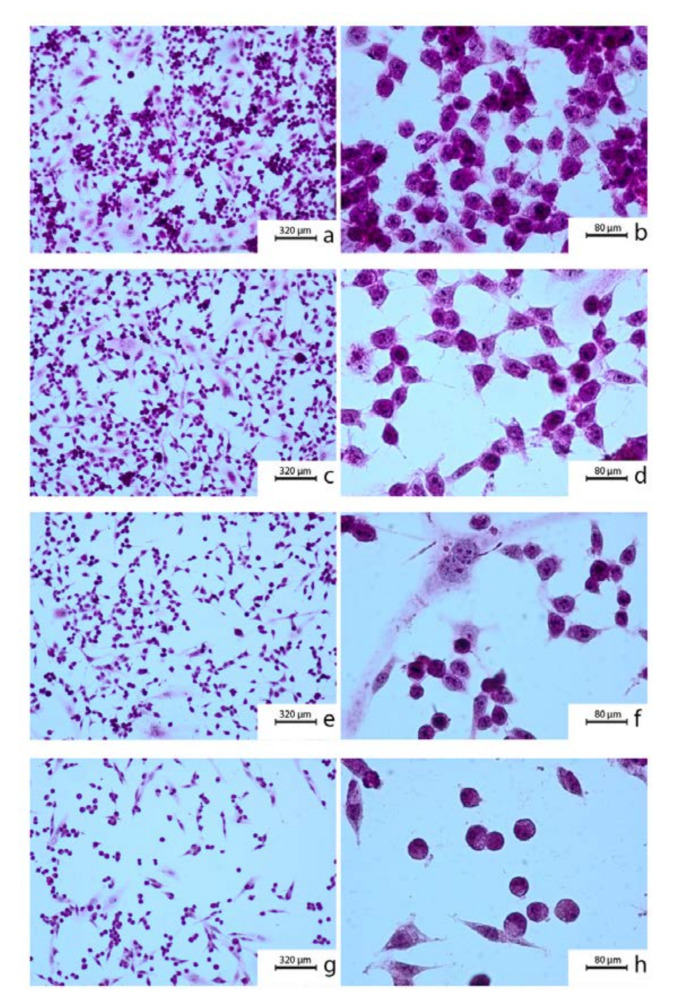
Cytomorphological view of OV7 cells after 24 h incubation: (**a**,**b**) control (no treatment); (**c**,**d**) 10 μM caffeic acid phenethyl ester (CAPE); (**e**,**f**) 50 μM CAPE; (**g**,**h**) 100 μM CAPE. To prepare the samples a hematoxylin and eosin staining was used. Exposition: optical magnification ×100 (**a**,**c**,**e**,**g**), ×400 (**b**,**d**,**f**,**h**). Main features: cellular polymorphism, hyperchromasia, long cytoplasmic protrusions (**a**,**b**); irregular nuclear shapes, numerous nucleoli visible, strong cellular adhesion (**c**,**d**); densification of the nuclei; shortening of cytoplasmatic protrusions (**e**,**f**); round cells, poorly attached to the surface (**g**,**h**).

In H-E staining, the OV7 cells revealed a large cell surface and nuclei whose contours and shapes were irregular. An increased nuclei/cytoplasmic ratio and significant nuclei discoloration were observed. In some cells, a large number of nuclei and their size with irregular outline were noteworthy. Some of the cells were multinucleated. Numerous subdivision figures were noted in the preparation. The use of CAPE at a concentration of 10 μM did not cause noticeable changes in the morphology of the cells tested. OV7 cells retained their size and irregular fusiform shape. Significant nucleus hyperchromia was observed. Strong cell-cell adhesion was noteworthy. Incubation of the OV7 cells with 50 μM CAPE resulted in a noticeable reduction in the number of cells in the preparation. Cells were smaller, cytoplasmic protrusions shortened, and cytoplasm thickened. Pleomorphism of the size, shape and color of cell nuclei were visible. After using CAPE at a concentration of 100 μM, a significant percentage of OV7 cells died and detached from the surface of the slide. Adherent cells had changed their size and shape. Attention was drawn to the disappearance of cytoplasmic protrusions and densification of the cytoplasm. Cell-cell contact was clearly weakened. These changes of CAPE treatment at the doses 0, 25 and 50 μM are shown in Figure 1.

### 2.2. Transmission Electron Microscope Visualization of OV7 Cancer Cell Line

**Figure 2:**
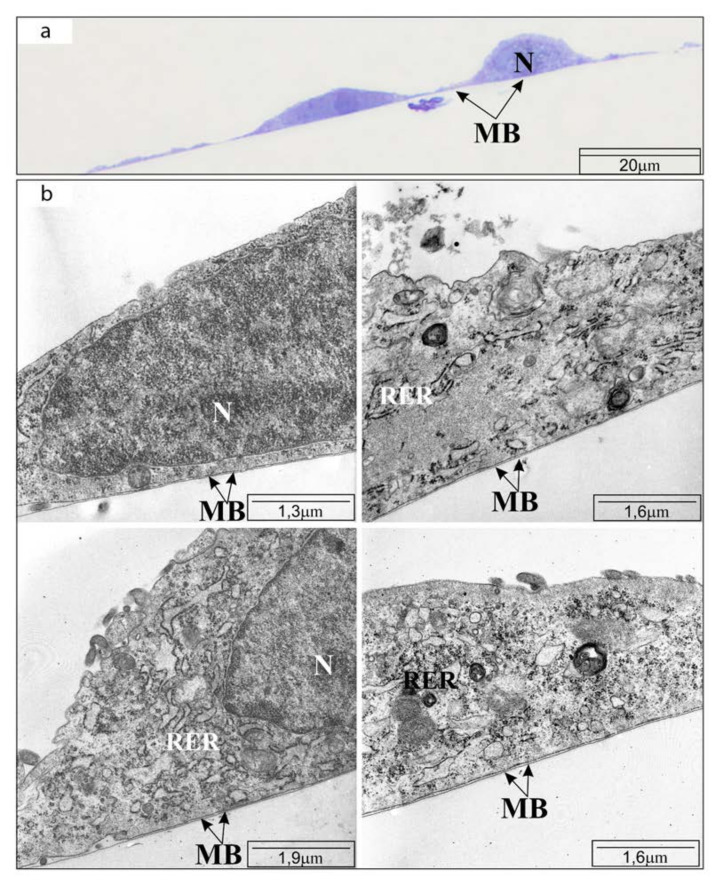
Transmission electron microscope visualization of OV7 cells not exposed to CAPE, after 24 h of incubation: (**a**) cross-section through the monolayer of cultured cells (half-thin sections, i.e., 0.5 µm thick); (**b**) cell organelles: MB—basement membrane; N—cell nucleus; RER—rough endoplasmic reticulum. Short description: cells tightly related to the ground, dense endoplasmic reticulum.

**Figure 3:**
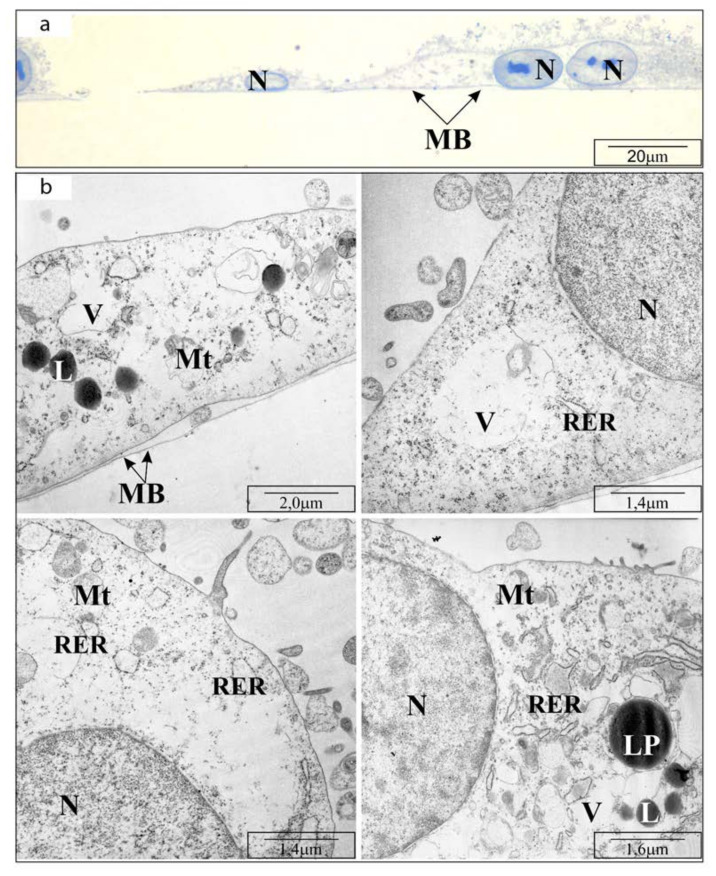
Transmission electron microscope visualization of OV7 cells treated with 25 µM CAPE, after 24 h of incubation: (**a**) cross-section through the monolayer of cultured cells (half-thin sections, i.e., 0.5 μm thick); (**b**) cell organelles: MB—basement membrane; N—cell nucleus; RER—rough endoplasmic reticulum; L—lysosomes; LP—lipoprotein bodies; Mt—mitochondria (highly deformed); V—vacuoles. Short description: cell enlargement, decrease in osmophilicity.

**Figure 4:**
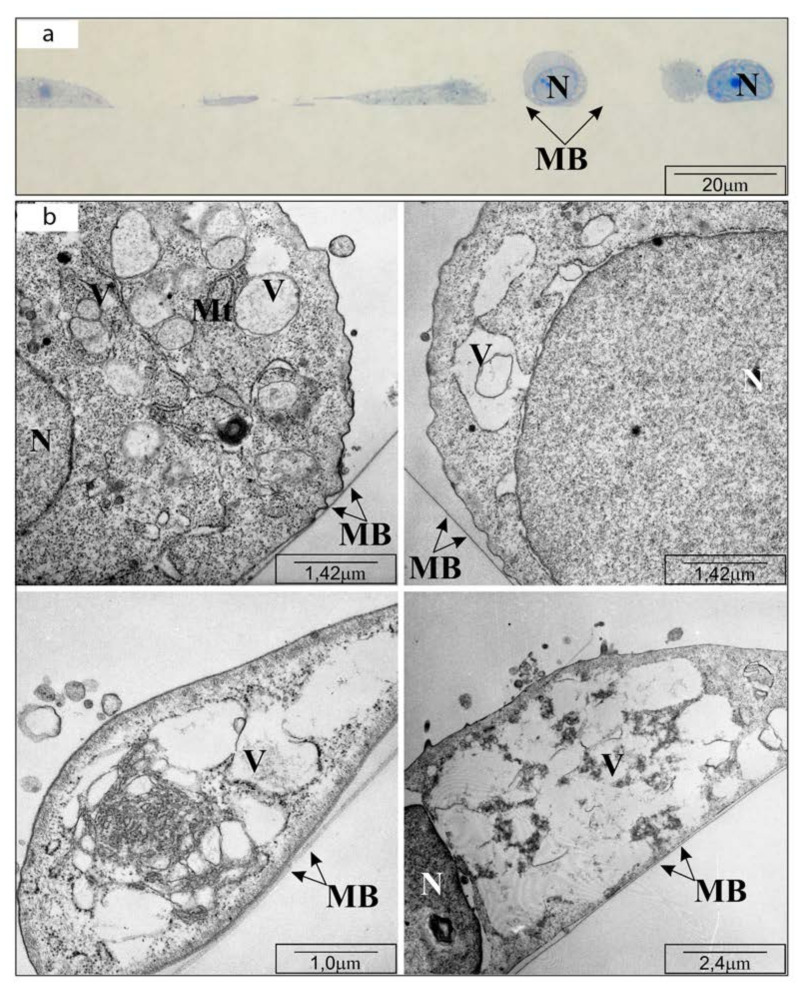
Transmission electron microscope visualization of OV7 cells treated with 50 µM CAPE, after 24 h of incubation: (**a**) cross-section through the monolayer of cultured cells (half-thin sections, i.e., 0.5 µm thick); (**b**) cell organelles: MB—basal membrane; N—cell nucleus; Mt—mitochondria; V—vacuoles. Short description: loss of cell adhesion, numerous vacuoles, matrix swelling.

In the control, cell imaging with TEM revealed flat cells, without granular inclusions but with osmophilic cytoplasm containing normal structure organelles. The arrangement of the cells was tight, the cells were connected by tight closing junctions. After using CAPE at a concentration of 25 μM, the cells were enlarged and their basal cytoplasm was clearly less osmophilic. The reduced osmophilicity of the cytoplasm was due to a reduction in the ”thinning“ of the basal cytoplasm proteins. After 24 h incubation with CAPE at a concentration of 50 μM, significantly fewer cells were observed on the basement membrane. The cells had elongated shapes and in many places showed poor adhesion to the ground-significantly protruded from the basal membrane. The snippets showed cells with a high degree of degeneration, i.e., cells with numerous membranes, whose arrangement could only suggest what organelles were building. The mitochondria showed signs of matrix swelling. The mitochondrial intermembrane spaces were widened and their structures were strongly distorted—only fragments of mitochondrial crests remained visible. The use of 100 μM CAPE resulted in the death of most cells tested, therefore single cells remaining in monolayers were not visible by TEM. The results are shown in Figure 2, Figure 3 and Figure 4, consequently.

### 2.3. Cell Viability by XTT-NR-SRB Assay

**Figure 5:**
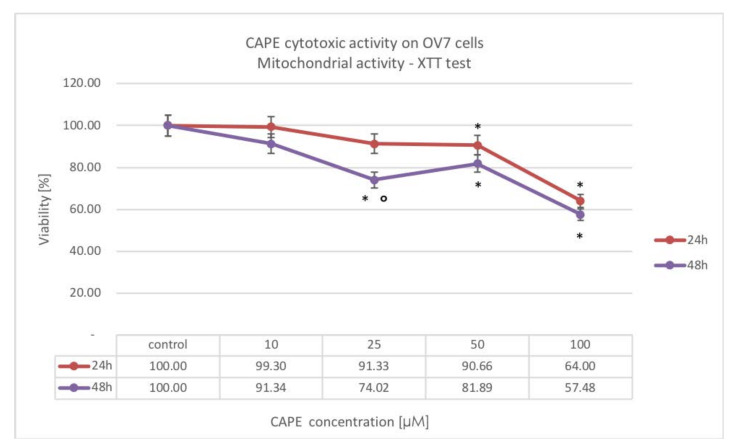
Cytotoxic effect of CAPE at concentrations from 10 to 100 μM at 24 h and 48 h incubation time against the OV7 ovarian cancer cells using the XTT assay; * statistically significant decrease in viability, relative to control (*p* < 0.05); ° statistically significant difference in cell viability after extending CAPE incubation from 24 to 48 h (*p* < 0.05).

A statistically significant decrease in the mitochondrial activity was observed after the use of CAPE at 50 and 100 μM concentrations, at 24 h, reaching 90.66% and 64.00%, respectively; while increasing the time to 48 h resulted in a significant cytotoxic effect also for CAPE at 25 μM dose: 74.02%. Only for CAPE at 25 μM dose, was there a significant distinction in viability between the 24 h (viability 91.33%) and the 48 h experiment, however, because by 25 μM CAPE at 24 h there was no significant difference from the control, we state there was no statistical difference in time domain in this experiment. The results are shown in Figure 5.

**Figure 6:**
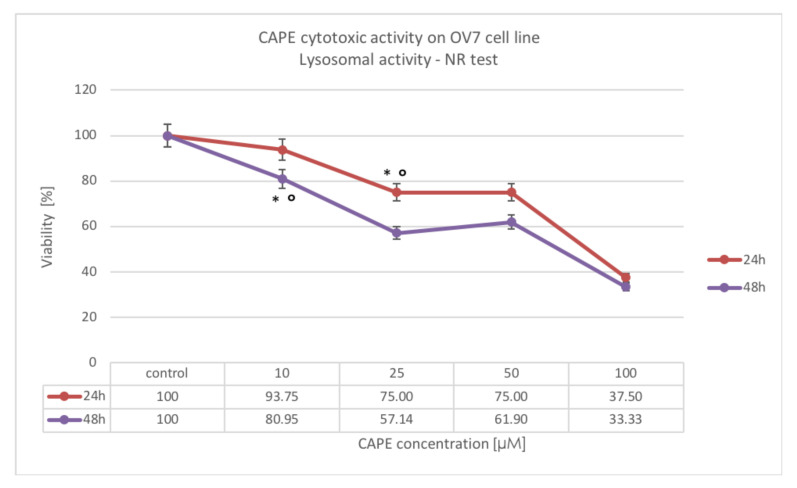
Cytotoxic effect of CAPE at concentrations from 10 to 100 μM at 24 h and 48 h incubation time against the OV7 ovarian cancer cells using the NR assay; * statistically significant decrease in viability, relative to control (*p* < 0.05); ° statistically significant difference in cell viability after extending CAPE incubation from 24 to 48 h (*p* < 0.05).

A statistically significant decrease in lysosomal activity was also observed after intoxication of CAPE with 100, 50 and 25 μM doses for 24 h. After 24 h incubation of OV7 cells with CAPE at a dose of 10 μM, no statistically significant decrease in viability was observed, while after the 48-h experiment, it was exhibited. The increase in the exposure time gave a statistically significant effect only for CAPE concentrations of 25 and 50 μM. Worth noting that for 10 μM of CAPE, after the experiment time was increased to 48 h, significant cytotoxic effect was also observed, while the 24-h result for this dose was not significant. The results are shown in Figure 6.

**Figure 7:**
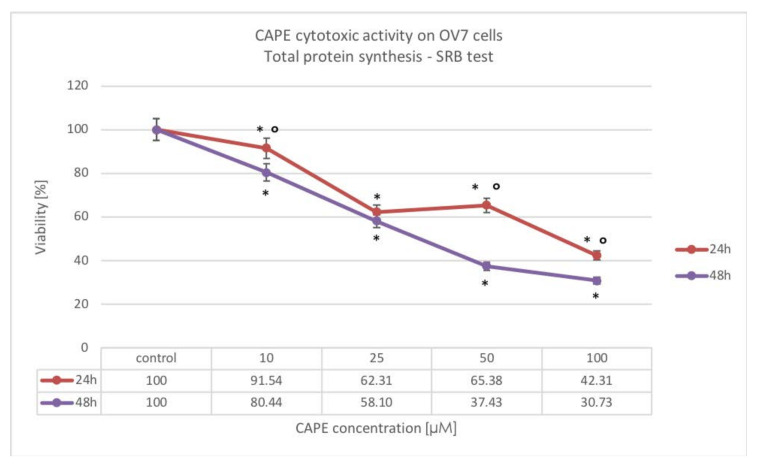
Cytotoxic effect of CAPE at concentrations from 10 to 100 μM at 24 and 48 h incubation time against the OV7 ovarian cancer cells using the SRB assay; * statistically significant decrease in viability, relative to control (*p* < 0.05); ° statistically significant difference in cell viability after extending CAPE incubation from 24 to 48 h (*p* < 0.05).

Our experiment showed a decrease in the total protein synthesis in OV7 cells intoxicated with CAPE. It was dependent on the concentration of tested compound and only for CAPE doses 50 and 100 μM on the incubation time. The strongest cytotoxic activity was determined for the highest tested CAPE concentration of 100 μM. A statistically significant increase in CAPE cytotoxicity over time was not distinguished, only at a dose of 25 μM. Figure 7 displays the results.

The half maximal inhibitory concentration (IC_50_) is a measure of the effectiveness of a substance in inhibiting viability, therefore for all performed XT-NR-SRB tests, the IC_50_ values were calculated and shown in Table 1.

### 2.4. Assessment of BAX/BCL2 Genes Expression by RT-PCR

During the experiment, the expression of the proapoptotic BAX gene and antiapoptotic BCL2 gene in ovarian OV7 cancer cells treated with CAPE were evaluated. Expression of both genes was significantly higher than in the control in every probe in dose dependent manner, for 24 and 48 h. Additionally, if checked in relation to time, increasing the observation time from 24 to 48 h resulted in significant growth of both gene expression, except for the highest, 100 μM dose of CAPE.

To compare evaluated gene expressions, the slope (gradient) analysis for both BAX and BCL2 was assessed. Slope function, based on linear regression, as a result gives the absolute value for single variable function. The steepness, incline, or grade of a line is measured by the absolute value of the slope. A slope with a greater slope value indicates a steeper line. The slope analysis is shown in Table 2. It was clearly visible, that the slope value was 10 times greater for proapoptotic BAX expression than for antiapoptotic BCL2 for both the 24 and 48 h experiments of OV7 cell treatment with CAPE.

**Figure 8:**
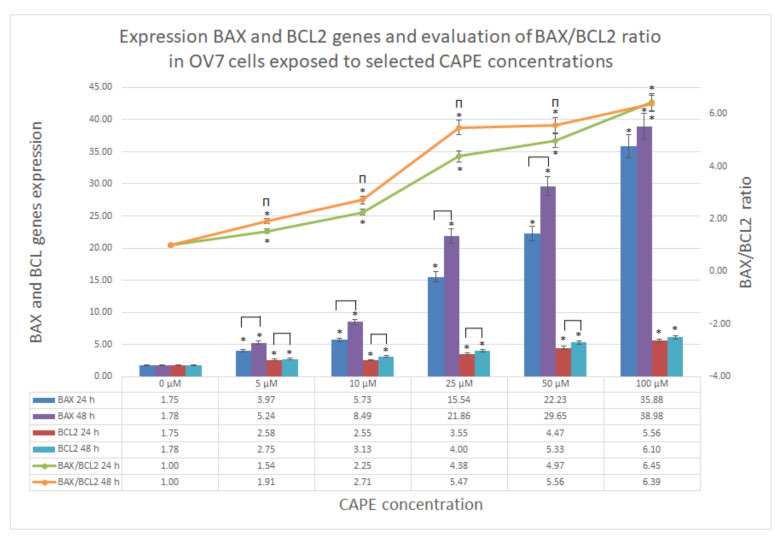
Expression of proapoptotic BAX and antiapoptotic BCL2 genes in ovarian OV7 cells exposed to CAPE at concentrations from 5 to 100 μM, at 24 and 48 h. Ratio of BAX/BCL2 > 1 displayed a dose-dependency and the rising advantage of proapoptotic gene expression BAX in relation to BCL2 antiapoptotic gene expression level; * statistically significant increase in expression levels of BAX, BCL2 and BAX/BCL2 gene expression ratio versus control (*p* < 0.05); Π statistically significant difference in expression levels of BAX, BCL2 and BAX/BCL2 gene expression ratio after extension of experiment incubation time from 24 to 48 h.

To assess CAPE proapoptotic activity, the ratio of BAX expression to BCL2 expression was calculated. Along with the increase in the concentration of CAPE and the extension of the experiment, an increase in the BAX/BCL2 ratio was observed. Only for 100 μM of CAPE, there was no significant difference in the time domain. Slope values of evaluated ratios shown in Table 2 did not exhibit significant difference in the time manner. This could be interpreted as the process represented by those genes expression (apoptosis) was stable in the time domain. All BAX/BCL2 results are shown in Figure 8.

### 2.5. Determination of Live, Necrotic, Early and Late Apoptotic Subpopulations Using an Accuri C6 Flow Cytometer

**Figure 9:**
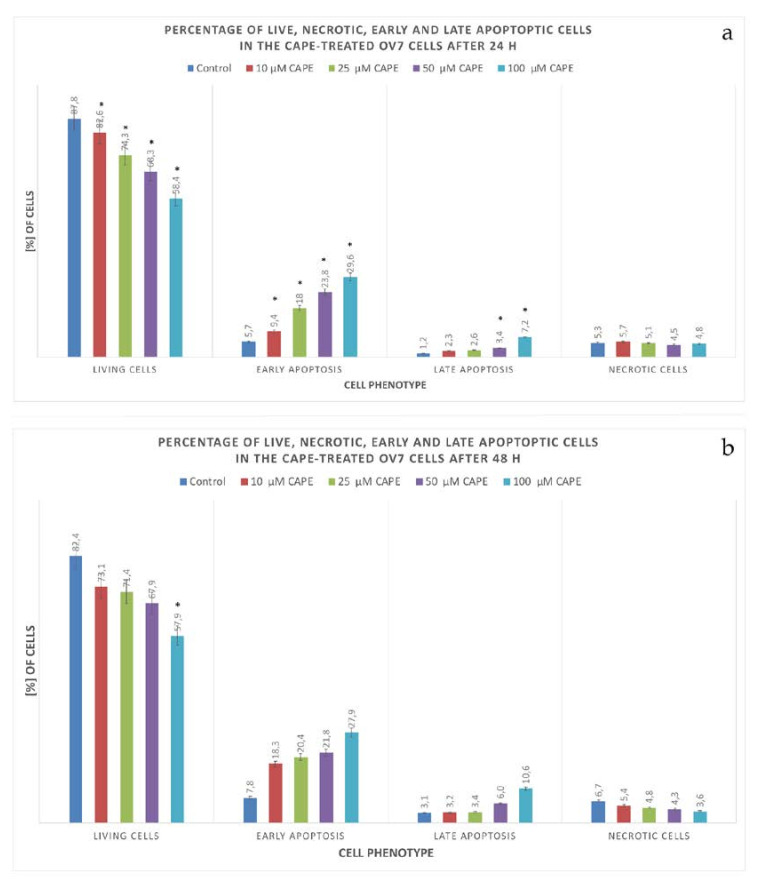
Apoptotic effect of selected CAPE concentrations on OV7 cells after (**a**) 24 h and (**b**) 48 h. Measurements using the V-FITC / PI assay; * statistically significant versus control (*p* < 0.05). The dose-dependent effect is visible for early apoptotic cells.

After 24 h of the experiment, a decrease in the number of viable cells was observed in each of the tested samples. The higher the concentration of CAPE used, the lower the percentage of viable cells noticed. OV7 cells treated with CAPE entered the process of apoptosis. The percentage of early apoptotic cells increased while CAPE concentration was increased. The results of apoptosis in OV7 cells treated with CAPE after 24 h are shown in Figure 9a.

After 48 h, the percentage of cells undergoing apoptosis increased compared to the results of the experiment finished after 24 h. The percentage of necrotic cells was comparable. The use of CAPE at 10 μM concentration resulted in a significant decrease in the percentage of living cells. This decrease was statistically significant relative to the control. Increasing concentrations of CAPE (25, 50 and 100 μM), caused a dose-proportional decrease in cell viability. With the increase in CAPE concentration, the number of living cells decreased, while the percentage of apoptotic cells increased. Compared to the control, a statistically significant increase in the number of cells undergoing apoptosis was demonstrated in each of the samples. The higher the CAPE concentration used, the higher the percentage of early-apoptotic cells. After 48 h of incubation, early apoptotic subpopulation dominated in OV7 cells. The 48 h results of CAPE on OV7 cells are shown in Figure 9b.

**Figure 10:**
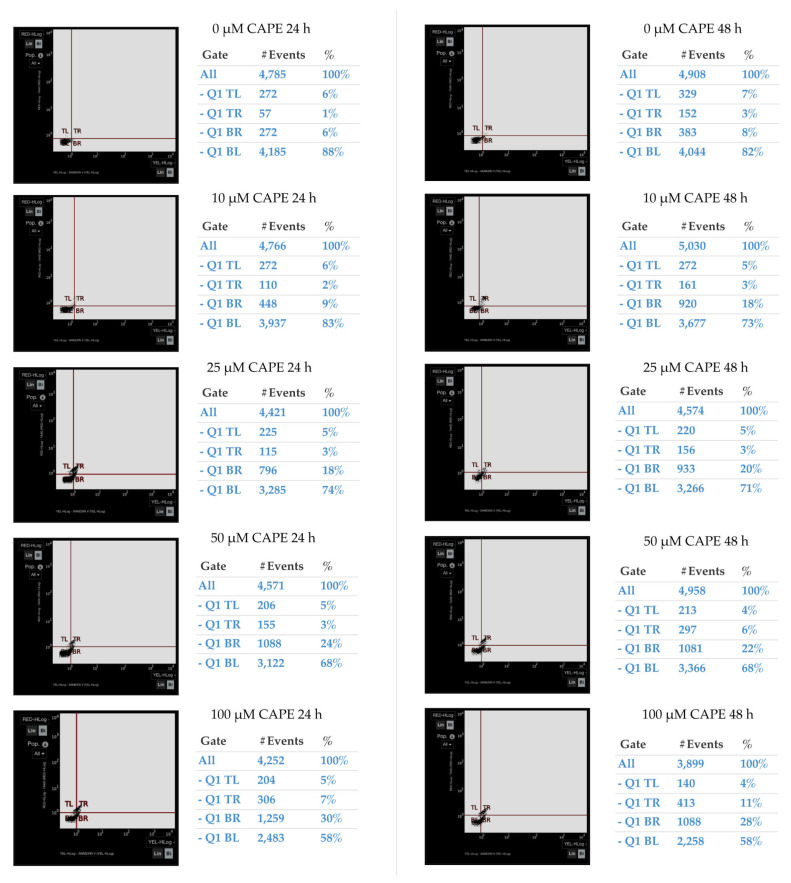
Representative plots showing the apoptotic effect of selected CAPE concentrations on ovarian cancer OV7 cells after 24 and 48 h. Necrotic cells are shown in top left (TL) quadrant, late apoptotic phenotype cells are in top right (TR) quadrant, early apoptotic phenotype cells are in bottom right (BR) quadrant, and in the bottom left (BL) quadrant there are cells with live phenotype. Measured with the V-FITC/PI assay.

Sample plots of apoptotic effect of CAPE on OV7 both after 24 and 48 h are shown in Figure 10.

## 3. Discussion

It is estimated that 60% of the drugs used in modern medicine come from plants. Research is underway to synthesize new, effective anticancer drugs that would be characterized by high bioavailability and low toxicity to healthy tissues [14,15].

Numerous literature reports suggest that one of the compounds that could be found useful in oncological therapy is CAPE. Caffeic acid derivatives have a number of beneficial biological activities, among which are antiviral and antibacterial, anti-inflammatory and immunomodulatory, as well as antioxidant and protective properties. CAPE has strong antiproliferative and cytotoxic activity on cancer cells, initiates apoptosis, reduces the synthesis of proinflammatory cytokines, inhibits angiogenesis, and enhances the effect of anticancer drugs. The concentrations of CAPE we used in the study were consistent with literature data and our previous experience [3,16,17,18,19,20].

The aim of this study was to assess the antiproliferative, cytotoxic and proapoptotic properties of CAPE against the OV7 serous ovarian cancer cell line.

The cytotoxic effect of CAPE on the OV7 cell line was visualized in H-E staining. The use of CAPE resulted in shrinking of tumor cells, shortening of their protrusions and thickening of the cytoplasm. The nuclei of the examined cells became pyknotic, it was difficult to see the nuclei structures, no division figures were observed.

A similar morphological picture of CAPE intoxicated cancer cells was observed by Rzepecka-Stojko et al. In their studies on triple-negative breast cancer lines MDA-MB-23 and Hs578T, comparable changes in cell morphology have been shown. After using CAPE at a concentration of 80 μM in the tested cell lines, a large percentage of cells, weakly adhering to the medium and with weakened intercellular contact, was noticed. The cytoplasm was concentrated, and the nucleus was dark. Compared to cells not exposed to CAPE, a decrease in pleomorphism was observed in the tested line [21].

Another breast cancer cell line was used in the study by El-Refaei et al. The ZR-75-1 cell line also proved to be sensitive to CAPE. After 24 h incubation, in tested cells vacuolization of the cytoplasm and numerous granulations in the area of the nucleus were noticed. The concentration of 20 μM CAPE resulted in cell detachment and the formation of cell aggregates [22].

In this study, changes in the morphology of OV7 cells were visualized using electron transmission microscopy for selected CAPE concentrations. It had been shown that under the influence of the tested compound, cells separated from the colonies, their structure became folded, and nuclear chromatin was strongly thickened and constricted the cell nucleus. Cytoplasmic swelling and perforation of cell membranes, observed in whole groups of cells, suggested osmotic imbalance, mitochondrial depolarization, and digestion of cellular structures by enzymes released from bursting lysosomes. This could be a typical picture of cell necrosis caused by extrinsic factors [23], however it could be also result of necroptosis. Necrosis is a highly adverse process for the body. By activating inflammatory processes and running in a chaotic manner, it can lead to the destruction of the entire organ or induction of a tumor process. Because the morphological picture of the OV7 cells shown in H-E staining, as well as other research methods used, suggest a rather proapoptotic effect of CAPE, there is a presumption that the observed changes can be associated with necroptosis [24,25]. This set us in position to plan new research to distinguish these processes.

The characteristic fragmentation of cell nuclei, swelling of mitochondrial crests and vacuolization of cytoplasm were observed in research by Mercantepe et al. in seminiferous tubules treated with cisplatin and CAPE synergistically [26]. Yang et al. also observed that in Ito cells (liver stellate cells of the HSC-T6 line) exposed to oxidative stress, administration of CAPE could lead to mitochondrial edema and the appearance of vacuolized cytoplasmic inclusions, however, these changes were not clearly expressed [27].

Necroptosis differs from apoptosis and autophagy in both its specific mechanism of action and physiological significance. In the morphological picture, necroptosis is manifested by swelling of cells and organelles, mainly mitochondria and early rupture of the cell membrane. The mechanism by which CAPE affects ovarian cancer cells is still under our investigation. Another experiment will allow us to assess which pathway regulating cell elimination is the target of the ester.

The obtained results have suggested that CAPE antiproliferative activity was mainly based on inhibition of cellular protein synthesis in OV7 cells. The lowest concentrations of the tested compound were needed to reduce the synthetic activity of OV7 cells, whereas inhibition of mitochondrial and lysosomal activity required the use of high doses of CAPE. Elongation the incubation time from 24 to 48 h for a CAPE concentration of 25 μM resulted in a statistically significant increase in inhibition of mitochondrial and lysosomal activity. Likewise, for a concentration of 50 μM CAPE, extending the experiment to 48 h increased the inhibitory effect on protein synthesis. The synthesis of nucleic acids, lipids and proteins is necessary for the proliferation and growth of cancer cells. The decrease in cell synthesizing activity suggested that the metabolism of OV7 cells slowed down and their mitotic potential decreased. Cell cycle testing would assess whether CAPE intoxicated OV7 cells did not stop at the G1 interphase phase—it is planned for the next stages of the experiment.

Concurrently, it is worth noting that CAPE does not completely suppress cellular protein synthesis. On the first day of the experiment, the synthesis activity of the cells tested remained at over 50% (except for the highest tested concentration of 100 μM CAPE), which may suggest that OV7 cells synthesized proteins necessary for apoptosis.

The effect of CAPE on ovarian cancer cell lines has also been studied by Gherman et al. The experiment evaluated the cytotoxic activity of the ester, relative to the cisplatin sensitive A2780 cells and the chemotherapy resistant A2780cis cell line. Low (<50 μM) CAPE concentrations were shown to significantly reduce cell proliferation and lead to activation of apoptosis in both cell lines [28], which is consistent with our observations.

In the experiment of Budisan et al., the cytotoxic effects of CAPE and kaemferol on the colorectal adenocarcinoma RCO and HTC-116 lines were assessed. In this study, examined cells appeared to be much more sensitive to CAPE than ovarian cancer cells. Effective IC_50_ values, calculated after 48 h incubation, were 36.87 μM for the RCO line and 3.33 μM for the HTC-116 line [29].

Studies on colon adenocarcinoma were also carried out by Tang et al. They showed that after 48 h incubation, the IC_50_ value for the HTC-116 line was 47.2 μM whereas, for the HT-29 line the minimum concentration inhibiting cell viability was 44.5 μM [30].

Higher CAPE doses were needed to inhibit the metabolic activity of OV7 serous ovarian cancer cells than other researchers have shown. This may be due to the high degree of malignancy of the cells tested. OV7 cells are poorly differentiated, the rate of xenobiotic penetration may be lower in them. The accessibility of target enzymes to CAPE, the ability of OV7 cells to activate repair processes and, above all, the ability to remove cytostatics from the cell by transport proteins could also affect the sensitivity of the cells tested—this requires further research.

An extremely interesting research was presented by Liu et al. who assessed the antitumor activity of CAPE both in vitro and in vivo. Using the SKOV3 serous adenocarcinoma cell line and an animal model infected with cancer cells, they showed that CAPE could inhibit ovarian cancer progression, reduce cell viability, migration and invasion. Inhibited expression of Ki67 (a marker of cell proliferation) and PCNA (proliferating cell nuclear antigen involved in replication, repair and regulation of the cell cycle) indicated a strong cytotoxic effect of CAPE. In contrast, weakened NF-κB phosphorylation leading to a decrease in proliferative activity and activation of apoptotic pathways confirmed that the mechanism of CAPE cytotoxicity was based on the induction of programmed death in cancer cells [31].

The therapeutic goal of treating oncological diseases is to induce apoptosis in cancer cells. Apoptosis, unlike necrosis, does not entail harmful consequences such as leakage of lysosomal enzymes into the extracellular environment and induction of a significant inflammatory reaction. Literature data indicate that CAPE has apoptosis-inducing effects on various cancer cells [19,32,33,34].

In the presented cytometric analysis, it has been observed that as CAPE concentration increased, the number of living OV7 cells decreased, while the percentage of apoptotic cells rose. After 24 h of the experiment, it was noticed that CAPE-treated OV7 cells began to enter the first stages of programmed cell death. The percentage of early apoptotic cells was higher in each tested sample than in the control. After 48 h of observation, the high percentage of OV7 cells just entering the path of programmed death indicated that CAPE was constantly acting on the tested cells. The percentage of necrotic cells at each of the tested concentrations was less than 10%. Of particular interest could be a strong cytotoxic and proapoptotic activity of the CAPE solution at a low, 10 μM dose. Literature reports suggest the effectiveness of rather higher doses of CAPE. The obtained result could be explained by the rapid stopping of NF-κB, which would require separate studies.

Similar results were published by Wu et al. Based on breast cancer of the MCF-7 and MDA-MB-231 cell lines, they confirmed that CAPE inhibited cell growth strongly affecting expression of genes and proteins. Dose-dependent increases in the synthesis of NF-κB nuclear factor inhibitor, cell cycle arrest, as well as increased apoptosis were observed in the study [35].

The purpose of the research by Chuu et al. was an assessment of the effect of CAPE on LNCaP 104-S, DU-145 and PC-3 prostate cell lines. By examining the percentage of mitotically active cells with a BD Facscan cytometer, the researchers have found that a dose of 3 μM CAPE, after 96 h incubation, inhibited the proliferation of tumor cells and stopped their cycle in the G1 phase. The number of cells in phase S decreased by half (from 30% in the control to 15% in the tested sample). The proapoptotic effect of CAPE was confirmed by assessing the expression of proteins regulating programmed cell death. The use of CAPE at concentrations from 20 to 80 μM caused a statistically significant decrease in the activity of genes encoding proteins from the IAP family (inhibitors of apoptosis). The most sensitive to CAPE was the LNCaP line. On the other hand, DU-145 cells were resistant to the effects of the ester [36].

Marin et al. also showed that multiple myeloma cells were sensitive to the antiproliferative and proapoptotic properties of CAPE. The action of CAPE was stronger in proportion to the increased concentration and duration of the experiment. Cytometric analysis showed that using CAPE at a dose of 50 μM causes apoptosis of 50% of the tested cells after 24 h. Proapoptotic activity has been associated with caspase-3 activation [37].

The presented data suggested that CAPE had strong proapoptotic activity that persisted throughout the experiment. This is extremely important because it may suggest that therapeutic CAPE concentrations persist in the tumor environment for a long time and can affect cells at various stages of the cell cycle. Research also suggests that CAPE can induce programmed cell death in several ways. Cancer cell arrest at the initial stages of cell cycle interphase, inhibition of transcription factor synthesis, and increased executive caspase activity may be potential targets for CAPE action in OV7 cells.

Studying the ratio of BAX/BCL2 genes, we found that the use of CAPE strongly enhances the activity of the proapoptotic gene in OV7 cells, while the expression of the gene synthesizing the antiapoptotic protein was also present, but 10 times lower. This can be justified by primary high resistance to any treatment. However, the results confirmed that CAPE induced apoptosis in OV7 cells, and specifically, CAPE dysregulated BAX/BCL2 balance in such a way that it activated proapoptotic BAX gene expression and simultaneously maintained antiapoptotic BCL2 gene expression at much lower levels. The results were dose and time (except 100 μM) dependent in both gene expressions. These results were consistent with other scientific reports.

To confirm the BAX/BCL2 results the Western blotting needs to be performed in order to quantify the targeted proteins. It is planned in further widen research, where also SRM (selected reaction monitoring) assay will be performed. Comparison of blotting and proteomic techniques would drive to multidimensional conclusions.

Early studies of CAPE-induced apoptosis mechanisms by Chen et al. have shown that this ester induced characteristic DNA fragmentation and morphological changes typical for apoptosis. In CAPE-treated HL-60 leukemia cells, caspase-3 was rapidly activated after 4 h, BCL2 expression decreased after 6 h and BAX expression intensified after 16 h [38].

The proapoptotic properties of CAPE against human myeloid leukemia U937 cells were studied by Jin et al. DAPI staining revealed that the typical apoptotic phenotype of cells (condensation, nuclear fragmentation) exposed to CAPE occurred after using a concentration of 5 μg/mL. CAPE led to the release of cytochrome C from mitochondrial structures, activation of caspase-3 and increased BAX gene expression, simultaneously silencing BCL2 activity. Studies have confirmed that the proapoptotic properties of CAPE were primarily based on mitochondrial activity, and could not be connected with death receptors (no Fas receptor activation) or signals from endoplasmic reticulum [39].

BAX activation, conformation change of the Bax protein and its transfer to the outer mitochondrial membrane were also observed in CAPE-treated head and neck cancer cell lines. Yu et al., examining the effect of the ester on YD15, HSC-4 and HN22 lines, showed that the proapoptotic properties of the compound could also be based on the activation of the Puma protein (p53 upregulated modulator of apoptosis). Puma had the ability to bind to all antiapoptotic proteins of the Bcl-2 family, so its activation enabled programmed death even in cells in which proteins inhibiting apoptosis were active. Moreover, the cytotoxic activity of CAPE was confirmed, and was depended in the time and dose domain [40].

Ulasil et al. proved in their study, that CAPE (as well as other substances contained in propolis, including thymoquinone and resveratrol) increased the activity of the p53 protein, caused an increase in BAX gene expression and inhibited the cell cycle in the G2 phase. A decrease in the activity of the NF-κB protein, lower expression of cyclin D1 and high activity of p21 protein, a strong inhibitor of cyclin dependent kinases, were observed in the experiment. A statistically significant increase in the percentage of apoptotic cells in the A549 lung adenocarcinoma cell line was observed after administration of 25 μM CAPE [41].

Assessment of proapoptotic protein concentration in the HT-29 cell line intoxicated by CAPE and its derivatives, described in the work of Tang et al., showed a significant increase in Bax, CytoC, P53 and P38 levels. Bax concentration increased by 0.7-fold relative to control, after using low doses of CAPE (in the range of 10–40 μM). This demonstrated CAPE’s ability to promote apoptosis in colorectal cancer cells. In addition, CAPE exhibited cell cycle inhibition properties. CAPE and CAPE-pNO2, by reducing the concentration of CDK2, might induce pRb dephosphorylation to promote cancer aging and stop the cell transition from the G1 to S phase. Moreover, examined compounds activated c-Myc regulation, inhibited telomere repair and prevented the immortalization of cancer cells [30].

A decreased BCL2/BAX ratio, together with increased caspase-3 activity and protein abundance were observed in PC-3, DU-145 and LNCaP prostate cancer cells after using CAPE synergistically with docetaxel and paclitaxel. Tolba et al. also proved that cyclin D1 and c-myc levels were significantly reduced in CAPE-chemotherapeutic treatment groups with a simultaneous increase in p27kip levels (cyclin-dependent kinase inhibitor 1B which controls the cell cycle progression at G1) [42].

This study shows that CAPE has antiproliferative, cytotoxic and proapoptotic effects on the OV7 serous ovarian cancer cells. This could have a potential use in developing new alternative therapeutic strategies or in addition to conventional cancer treatment regimens [43]. The results of the study push us towards the next research that will compare the effect of CAPE on different types of ovarian cancer lines and noncancerous ovarian cells. However, important aspects regarding in vivo effects such as dose effectiveness, effects on healthy cells, molecular changes, bioavailability and long-term exposure must be considered before formulating consistent therapeutic strategies.

## 4. Materials and Methods

### 4.1. Cell Lines and Reagents

#### 4.1.1. Ovarian Cancer Cell Line

The research was carried out on the OV7 serous cancer cell line from Sigma Aldrich (Warsaw, Poland, ref. No: 96020764). Based on manufacturer information, the OV7 cell line was established from solid ovarian tumor tissue taken from a 78-year-old female patient. The tumor was characterized as stage III, mixed tumor type and poorly differentiated.

The OV7 cell culture was carried out according to the manufacturer’s instructions, at 37 °C and 5% CO_2_ atmosphere, using the Panasonic MCO-170AICUV incubator (Oizumi-Machi, Japan). The culture was carried out on DMEM: HAMS F12 (1:1) medium, with the addition of 5% bovine serum (FBS Fetal Bovine Serum; from PAA Laboratories, Pasching, Austria) and supplemented with 2 mM glutamine, 10 μg/mL insulin and hydrocortisone in concentration 0.5 μg/mL. A composition of antibiotics was added to the medium at the following concentrations: 100 IU/mL of penicillin, 100 μL/mL of streptomycin and 250 μL/L of amphotericin B. The cells were grown in 25 cm^2^ bottles and passaged after reaching 80% confluence.

#### 4.1.2. CAPE 

The CAPE used in the study was extracted by the manufacturer Sigma Aldrich (Warsaw, Poland, ref. No: C8221) from propolis, using the HPLC (high-performance liquid chromatography) method. CAPE used in the experiment, according to manufacturer data had ≥ 97% purity. It is soluble in DMSO and ethanol.

### 4.2. Hematoxyin-Eosin Staining

After trypsinization, the OV7 cells were inoculated onto Nunc Lab-Tek II Chamber Slide System four-chamber culture slides (supplier Thermo Fisher, ref. No. 154526PK Rochester, NY, USA) at a count of 1000 cells/well. A sample of 1 mL of pure medium was injected into each chamber. After a 24 h incubation period, the cells clogged on the surface of the slide and the confluence was estimated to 80%. The proper concentrations of CAPE (10, 50 and 100 μM) were prepared in the fresh culture medium and inserted into each of the chambers. Cultivation was continued for another 24 h in standard conditions.

After that, the chambers were removed, and the cells were fixed on the surface of the slide by 12-h incubation in 96% ethyl alcohol. The fixed cells were hydrated in a decreasing alcohol series (99.6%; 96%; 90%; 80%; 70%; 50%) and stained for 12 min in a hematoxylin solution. Then, the slides were washed under tap water until the blue color was visible. Next, the cells were incubated in eosin solution for 30 s and washed with PBS solution. After that, the cells were dehydrated in a growing alcohol series (50%; 70%; 80%; 90%; 96%; 99.6%) and immersed in the ethanol and xylene mixture (50:50) for 1 min. Finally, the slides were put in pure xylene for 1 h and then closed with a coverslip using Canadian lotion.

The morphology of OV7 cells was obtained using Zeiss Axiostar microscope (Carl Zeiss, Jena, Germany).

### 4.3. Transmission Electron Microscope Visualization

The OV7 cells were inoculated into six-well adherent culture plates and supplemented with medium until 80% confluence. The supernatant medium was then decanted and selected CAPE solutions (25, 50, 100 μM) were inserted into the wells. Control cells were protected with fresh medium containing no tested compound.

After 24 h incubation, the medium was gently removed from the plates, therefore 3 mL/well of 2.5% paraformaldehyde in phosphate buffer was injected. The material was fixed for 24 h at 2–8 °C and then inverted at increasing concentrations of ethanol. After the first fixation, secondary fixation was performed, involving osmium oxide IV ions. Then, the tested cells were again dehydrated in alcohol solutions of increasing concentrations, and in the next stage, the preparations were embedded in the epon mixture (Epon 812 Polyscience).

The obtained blocks were cut into snippets using a LEICA ultra-microtome with a glass and a diamond knife. Sections 20 to 60 nm thick were evaluated in a transmission microscope. Half-thin sections were stained with an aqueous solution of methylene blue and observed under a light microscope. Ultrathin sections (50–80 nm) were precisely placed on 3mm diameter copper meshes and fixed in uranyl acetate and lead citrate.

Cell visualization was assessed using a high-resolution transmission electron microscope (TEM) Hitachi H500 (Hitachi Ltd., Tokyo, Japan) at 75 kV.

### 4.4. XTT-NR-SRB Assay

The XTT-NR-SRB test (Xenometrix; supplier TIGRET Sp. z o.o., Warsaw, Poland ref. No. PAN I 96.1200) is a complex colorimetric test that is used to evaluate mitochondrial metabolism, integrity and activity of lysosomes, and a factor of the total cell protein synthesis in response to intoxication with a tested compound. The kit allows sequential measurement of three cytotoxicity parameters in one cell culture. This test was carried out strictly according to the procedure described by the manufacturer. CAPE was used at concentrations of 10, 25, 50, or 100 μM, with 24 and 48 h incubation period. Accordingly, data were normalized and expressed as % of viability over controls.

#### 4.4.1. Cell Viability by Mitochondrial Activity Assay (XTT)

XTT (2,3-bis[2-methoxy-4-nitro-5-sulfopheny]-2*H*-tetrazolium-5-carboxyanilide inner salt) tetrazole salts are reduced to formazan by the action of mitochondrial dehydrogenase, an enzyme which is active only in cells with intact metabolism and the respiratory chain. The amount of formazan is calculated photometrically and correlates with the number of viable cells. Enzyme activity was measured at 480 nm.

#### 4.4.2. Cytototoxicity by Lysosomal Activity Assay (NR)

Neutral red (NR) is a weak cationic dye that quickly penetrates the cell membrane and accumulates inside the cell in lysosomes (lysosomal pH < cytoplasmic pH). Changes in the cell surface or sensitization of the lysosomal membrane lead to lysosomal fragility and other changes that slowly become irreversible. Such changes caused by xenobiotics result in reduced uptake and binding of NR. Thus, a living, damaged or necrotic cell can be distinguished. The amount of bound dye in the cells is measured spectrophotometrically at 540 nm and it is directly proportional to the number of cells with intact membrane.

#### 4.4.3. Cell Proliferation by Total Protein Synthesis Assay (SRB)

Sulforhodamine B (SRB) is an anionic dye that binds to proteins with electrostatic bonds. The fixed SRB dye is measured photometrically (at λ = 540 nm) after dissolution and correlates with the rate of total protein synthesis, and thus with cell proliferation.

### 4.5. BAX/BCL2 Genes Expression by qRT-PCR

QuantiFast Probe RT-PCR test was used to assess the expression of BCL2 and BAX genes. The assay was performed according to the procedure prepared by the manufacturer (Qiagen, Germany, ref. No.: 204454).

Cells to be tested were grown in standard conditions on 6-well plates. After achieving the desired confluence, the medium from the culture was gently poured and CAPE solutions were injected into the cells at concentrations of 5, 10, 25, 50 and 100 μM. After 24 h/48 h incubation, the culture medium was gently removed and cells were separated from the medium by trypsinization. Then, the cells were centrifuged (5 min at 1,500 rpm) and the obtained pellet was suspended in 10 mL of Trizol, then incubated for 30 min at room temperature. The solution was transferred to RNase-free tubes and 2 mL of chloroform was added to every tube. Each sample was then vigorously stirred for 15 s. After 2–3 min incubation at room temperature, the tubes were centrifuged for 20 min at 5000 rpm and at 4 °C. The supernatant was then mixed with 5 mL isopropane alcohol and incubated at room temperature for 5–10 min. After the next centrifugation, the samples were decanted and the pellet was dissolved in 10 mL 70% ethyl alcohol. Next, 600 μL of water with DEPC (diethylpyrocarbonate) was added to each sample to dissolve RNA and the samples were incubated on ice for 10 min. Then, the material was transferred to 65 °C and incubated for 5 min. Isolated RNA was stored at −80 °C until the next steps of research. After preparation of the reaction mixture, an RNA template (100 ng/reaction) was added to individual wells of the PCR plate and the qRT-PCR cycler was programmed.

In order to determine the concentration of the tested sequence in individual samples, a relative method was used. It is based on expressing the concentration of the sequence in samples unknown as multiples of its amount in the reference sample, i.e., in the calibrator.

The comparison of unknown samples with the calibrator is usually preceded by normalization of the level of gene expression, which is carried out to correct the differences between the compared samples. Normalization can be performed by duplicating the control gene next to the test gene. In the experiment, the gene encoding GAPDH (glyceraldehyde 3-phosphate dehydrogenase) was recognized as a control gene.

The relative method (also: comparative) is based on a mathematical model that allows the calculation of the relative differences in the level of expression of the studied gene between the tested samples and the reference sample.

In the first stage of the experiment, threshold cycles (Ct) of the replication reactions of the examined and control genes were determined. Then, for individual samples, the differences between the Ct values on the test gene and the control gene (ΔCt) are calculated. The calculation is carried out for both tested and calibration tests:

ΔCt (test sample) = Ct (target gene) − Ct (reference gene),(1)

ΔCt (calibrator) = Ct (target gene) − Ct (reference gene).(2)

In the next stage, ΔΔCt is calculated for each of the samples:

ΔΔCt = ΔCt (test sample) − ΔCt (calibrator)(3)

The calculation of the normalized value of the relative level of expression of the test gene in the test sample relative to the calibrator is based on the formula:

R = 2^−ΔΔ Ct^, (4)

The value of parameter R equal to 1 indicates that the level of expression or the number of copies of the gene in the calibration and the test sample are the same. The R < 1 indicates a lower expression level in the sample comparing to the calibrator. Whereas the value R > 1 suggests a higher gene expression in the tested sample as compared to the reference sample.

### 4.6. Annexin V FITC and Propidium Iodide Staining for Cytometry Analysis

The OV7 cells were cultured on a six-well plate and incubated with growth medium until 80–90% confluency was reached (approximately 250,000 cells per well). The cells were then supplemented with a complete culture medium containing 10, 25, 50 and 100 μM of CAPE and left for incubation of 24 and 48 h. In the next step, 1 mL of acutase was added to each well and the probes were centrifuged for 5 min at 1500 rpm to obtain a precipitate. After obtaining a pellet, the cells were washed and dissolved in 1mL binding buffer. Then, 100 μL of cell suspension was pipetted into Eppendorf and 5 μL FITC Annexin V and 5 μL PI were added. The apoptosis was determined with a BD Accuri C6 flow cytometer. The plots were generated by Red Matter software (Redmatterapp v. 1.0.0., Red Matter, Dublin, Ireland).

### 4.7. Statistical Analysis

All results are expressed as means ±SD obtained from three separate experiments and performed in quadruplicates (n = 12). The results were performed with independent sample t-tests. The experimental means (each one separately) were compared to the means of untreated cells (control) harvested in a parallel manner for 24 and 48 h, respectively. Moreover, for the same concentrations, we compared the differences for 24 and 48 h time of incubation. For significance, differences were tested for significance using the one- or multiple-way Levene’s and Friedman‘s ANOVA tests. Moreover, for BAX and BCL2 evaluation, the slope analysis was performed. A p-value less than 0.05 was considered statistically significant.

## 5. Conclusions

CAPE has antiproliferative and cytotoxic activity on OV7 serous ovarian cancer cell line. The cytotoxic activity of CAPE depends on its concentration and incubation time with the OV7 cells. CAPE shows the strongest action relative to the total synthesis of cellular proteins, while it exhibited a limited effect on the mitochondrial activity of the OV7 cells. Presented results in this study can suggest that aggressiveness in serous ovarian tumors (for the first time in OV7 cells) in addition to proliferation, could be linked also to apoptosis-related constituents; specifically, CAPE dysregulated BAX/BCL2 balance activating BAX gene expression level 10 times higher than BCL2. In further research, we plan in vivo research with a xenograft model to investigate the anticancer effect of CAPE on ovarian cancer. Moreover, investigation of liberation, absorption, distribution, metabolism and excretion (LADME) and therefore, the optimal doses and form of administration seem to be reasonable to examine and assure best bioavailability.

## Figures and Tables

**Table 1:** IC_50_ (µM) values of CAPE activity on OV7 cells by various tests.

Experiment Time	XTT	NR	SRB
24 h	142.58 ^1^	81.43	80.08
48 h	128.44 ^1^	64.55	49.57

^1^ Exceeded tested concentrations.

**Table 2:** Slope (gradient) analysis for BAX and BCL2 expression values on CAPE treated OV7 cells.

Gene/Time	Slope Value
BAX/24 h	0.342
BCL2/24 h	0.0356
BAX/48 h	0.371
BCL2/48 h	0.040
BAX/BCL2 24 h	0.053 ^1^
BAX/BCL2 48 h	0.050 ^1^

^1^ Not significantly different.
